# Limited Immunogenicity of an HLA-A*03:01-restricted Epitope of Erv-k-env in Non-hiv-1 Settings: Implications for Adoptive Cell Therapy in Cancer

**DOI:** 10.21203/rs.3.rs-4432372/v1

**Published:** 2024-05-30

**Authors:** Erin E. Grundy, Lauren C. Shaw, Loretta Wang, Daniel J. Powell, Mario Ostrowski, R. Brad Jones, C. Russell Y. Cruz, Heather Gordish-Dressman, Catherine M. Bollard, Katherine B. Chiappinelli

**Affiliations:** George Washington University; University of Pennsylvania; George Washington University; University of Pennsylvania; University of Toronto; Weill Cornell Graduate School of Medical Medical Sciences; Children’s National Hospital; Children’s National Hospital; Children’s National Hospital; George Washington University

**Keywords:** Tumor immunology, Immunotherapy, Repetitive elements, Endogenous retroviruses, T cell receptor

## Abstract

Repetitive elements (REs) are often expressed at higher levels in tumor cells than normal cells, implicating these genomic regions as an untapped pool of tumor-associated antigens. In ovarian cancer (OC), protein from the RE ERV-K is frequently expressed by tumor cells. Here we determined whether the targeting of a previously identified immunogenic epitope in the envelope gene *(env)* of ERV-K resulted in target antigen specificity in non-HIV-1 settings. We found that transducing healthy donor T cells with an ERV-K-Env-specific T cell receptor construct resulted in antigen specificity only when co-cultured with HLA-A*03:01 B lymphoblastoid cells. Furthermore, these transduced T cells were not specific for HLA-A*03:01 + OC cells nor for the cognate peptide in HLA-matched systems from multiple healthy donors. These data suggest that the ERV-K-Env epitope recognized by this T cell receptor is of low immunogenicity and has limited potential as a T cell target for OC.

## BACKGROUND

Due to the lack of curative therapies for ovarian cancer (OC), patients with this disease are typically diagnosed at late stages and often succumb to this malignancy [1] [2]. Unlike the dramatic extensions in survival rates reported following the administration of immunotherapy in more immunogenic cancer types, such as melanoma and non-small cell lung cancer, OC is less responsive to immune checkpoint blockade [3] [4] [5] [6] [7] [8] [9] [10]. While OC does not respond well to immunotherapy, treatment of tumor cells with epigenetic inhibitors stimulates an anti-tumor interferon immune signaling response in *in vitro* and murine models of OC [11] [12] [13]. For example, treatment of tumor cells with DNA methyltransferase inhibitors or histone deacetylase inhibitors induces the expression of repetitive elements (REs)—genomic regions that are epigenetically silenced in healthy cells to prevent their mobility and destabilization of the genome [14] [15]. As the structure of some transcribed REs resembles that of viral nucleic acids, their expression stimulates type I and III interferon signaling pathways in tumor cells in a process termed “viral mimicry” [11] [16] [17]. Treatment of tumor cells with epigenetic inhibitors also increases the expression of antigen processing and presentation machinery, rendering tumor cells more visible to the immune system [18]. As most REs do not encode functional proteins[19] [20], their increased expression in response to epigenetic therapy makes them ideal targetable tumor-associated antigens.

A higher number of intratumoral T cells is positively correlated with higher overall survival in patients with OC, implying that the presence of T cells within the tumor is associated with controlling disease long-term[21] [22]. While OC development is driven by genomic mutations, it has a relatively low mutational burden, with few neoantigens present [5] [23] [24]. One reason that patients with OC who have higher numbers of intratumoral T cells experience longer overall survival rates may be that these intratumoral T cells are recognizing non-conventional antigens, derived from REs. Long terminal repeats are one class of REs frequently overexpressed in OC [25] [26] [27]. Endogenous retroviruses (ERVs)—a type of long terminal repeat—are the remains of viruses that have integrated into the human germline millions of years ago [28] [29]. ERVs are composed of three genes—gag, *pol*, and *env—*with a long terminal repeat flanking the genes. Protein derived from the *gag* and *env* genes of the RE family ERV-K has been shown to be frequently expressed in several types of cancers, including OC [30] [31] [25] [32] [33] [34] [35].

Previous work has shown that protein derived from the envelope gene of the ERV-K(HML-2) group (ERV-K-Env) is immunogenic in T cells expanded from peripheral blood mononuclear cells (PBMCs) derived from patients with OC [27].Separately, a CD8 + T cell clone that recognizes an epitope derived from ERV-K-Env has been isolated from an individual infected with human immunodeficiency virus-1 (HIV-1) [36]. The minimal epitope recognized by this clone was mapped to an HLA-A*03:01-restricted epitope of ERV-K-Env that is 12–15 amino acids in length. This peptide spans amino acids 413–427 of the *env* gene, placing it in the surface domain [37] [38]. As HLA I (human equivalent of major histocompatibility complex I/MHC I) canonically presents epitopes that are 8–11 amino acids in length, a 12–15 amino acid peptide is unusually long for HLA I to present to CD8 + T cells[39] [40]. However, this ERV-K-Env-specific T cell clone eliminated HLA-matched CD4 + T cells infected with HIV-1. For this study, we aimed to determine whether this HLA-A*03:01-restricted TCR clone could recognize its cognate epitope outside the HIV-1 setting, with the goal of using it as an immunogenic OC target. As some populations in the United States have up to 6% HLA-A*03:01 positivity, this may be a fairly broadly applicable OC treatment target [41]. In this work, we used IFN-γ enzyme-linked immunosorbent spot (ELISpot) assays and intracellular cytokine staining to determine that T cells transduced with the ERV-K-Env-specific TCR only responded to the cognate peptide when presented by HLA-A*03:01 B lymphoblastoid cells from multiple donors, but not HLA-A*03:01 OC cells or HLA-matched antigen presenting cells. Together, our findings suggest that the epitope is of low immunogenicity in non-HIV-1 settings and not an immunogenic tumor-associated antigen.

## RESULTS

OM9.2 T cells did not respond to HLA-A*03:01 OC cell lines more than untransduced T cells. The HLA-A*03:01-restricted ERV-K-Env-specific TCR was first identified in an individual positive for HIV-1, donor OM9 [36]. The epitope sequence of ERV-K-Env that the TCR recognizes is shown in **Figure S1A.** The minimal epitope is 12 amino acids in length, which is unusually long for MHC class I [40]. The longer epitope (the 15mer) is identical to the 12mer with 3 additional amino acids at the C-terminus end of the epitope. Hereafter, the epitopes are referred to together as ERV-K-Env. The first plasmid construct of this TCR (construct OM9.1), yielded low transduction efficiency in T cells isolated from several healthy donors (average ~ 12.1% ± 9.1%) (**Figure S1B-D**). The construct was optimized (construct OM9.2) by cloning the TCR into a lentiviral vector under the control of the EF-1 α promoter (**Figure S2A-B**). To confirm that the TCR was being expressed on the cell surface, SupT1 cells—which lack an endogenously expressed TCR on their surface—were transduced with the OM9.2 construct and analyzed for CD3 and TCR-α/β expression via flow cytometry (**Figure S2C**). Following this validation, GFP was used to confirm the transduction efficiency of donors in subsequent experiments. The OM9.2 construct yielded an average transduction efficiency of ~ 83.9% ± 12.5% in T cells isolated from multiple healthy donors (Figure S2D), indicating that primary T cells can be efficiently transduced to express the TCR.

**Figure 1:**
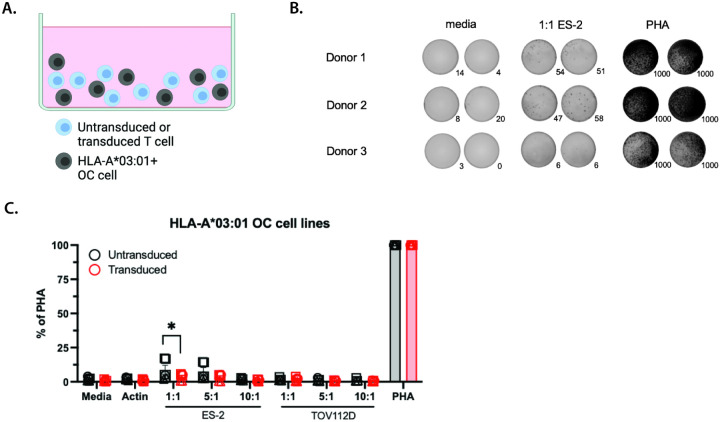
OM9.2 T cells did not respond to HLA-A*03:01 OC cells more than untransduced T cells. A. Diagram of the ELISpot assay design used in B-C. B. Representative images of developed wells for the level of background IFN-γ secretion (media), IFN-γ secretion in the presence of ES-2 cells (1:1 ES-2), and positive control of IFN-γ (PHA) for the ELISpot assay quantified in C. Number of spots in each well are indicated to the bottom right of each image. Images shown are for OM9.2 T cells. C. Number of IFN-γspots per well normalized to the positive control (expressed as % of PHA) of untransduced T cells (black) and OM9.2 T cells (red) for the indicated conditions: media = background level of IFN-γ secretion from T cells, actin = negative control, various effector:target ratios of T cells (effectors) with HLA-A*03:01 OC cell lines (targets), PHA = positive control. Each donor is indicated by a different symbol. ELISpot conditions were plated in duplicate or triplicate as cell numbers allowed for n = 3 donors. Error bars represent SEM of all data points for all donors. The Wilcoxon rank sum test was used to compare the number of IFN-γ spots between untransduced and transduced T cells (indicated by *). The Kruskal Wallis test was used to compare the number of IFN-γ spots between each antigen condition for untransduced or transduced cells. Any Kruskal Wallis test that had p < 0.05 was followed by post-hoc pairwise Wilcoxon rank sum tests and adjusted for multiple comparisons (indicated by #). All statistical comparisons are in Tables S1 and S2. *p < 0.05, **p < 0.005, ***p < 0.0005. ELISpot = Enzyme-linked immunosorbent spot; PHA = phytohemagglutinin; OC = ovarian cancer; SEM = standard error of the mean.

To test whether OM9.2-expressing T cells would respond to HLA-A*03:01 OC cells at an increased level compared to unmodified T cells, we used the enzyme-linked immunosorbent spot (ELISpot) assay to detect interferon-γ (IFN-γ) secretion from T cells in the presence of antigen (Fig. 1A). Both untransduced and OM9.2 T cells had low background secretion of IFN-γ when cultured in the absence of any antigen or with a negative control peptide (Fig. 1B–C, “media” and “actin”, respectively; p-values for all comparisons for all figures in **Tables S1 and S2**). There was no significant difference in the amount of IFN-γ secreted by OM9.2 T cells compared to untransduced T cells plated with HLA-A*03:01 OC cell lines at any effector:target ratio tested (Fig. 1B–C, “ES-2” and “TOV112D”). However, both OM9.2 T cells and untransduced T cells were immunologically functional as they were both capable of secreting large amounts of IFN-γ in the presence of the mitogen phytohemagglutinin (PHA), which stimulates T cells independent of TCR specificity [43] (Fig. 1 B–C, “PHA”).These data indicate that expression of the HLA-A*03:01-restricted ERV-K-Env-specific TCR does not allow OM9.2 T cells to respond to HLA-A*03:01 OC cells at a higher level compared to untransduced T cells.

### OM9.2 T cells were not activated by free ERV-K-Env peptide.

**Figure 2:**
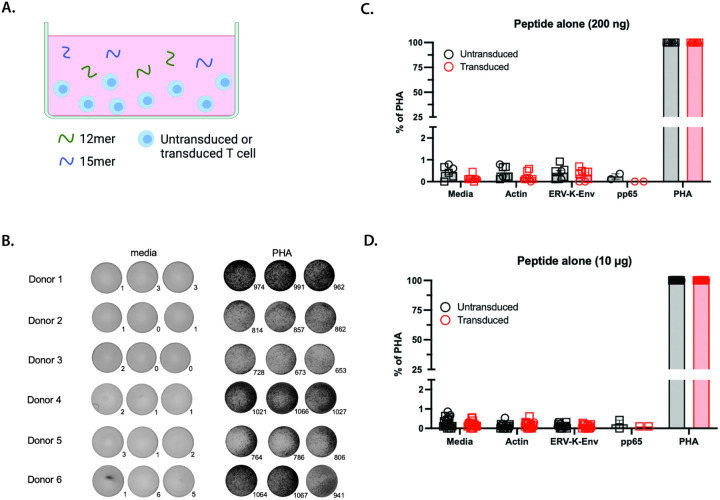
OM9.2 T cells were not activated by free ERV-K-Env peptide. A. Diagram of the ELISpot assay design used in B-D. B. Representative images of developed wells for the level of background IFN-γ secretion (media) and positive control of IFN-γ(PHA) for the ELISpot assays quantified in C-D. Number of spots in each well are indicated to the bottom right of each image. Images shown are for OM9.2 T cells. C-D. Number of IFN-γ spots per well normalized to the positive control (expressed as % of PHA) of untransduced T cells (black) and OM9.2 T cells (red) for the indicated conditions: media = background level of IFN-γ secretion from T cells, actin = negative control, ERV-K-Env = ERV-K-Env peptide, pp65 = HLA-B*35 off-target control, PHA = positive control. Each donor is indicated by a different symbol. ELISpot conditions were plated with the indicated peptide concentrations in duplicate or triplicate as cell numbers allowed at 200 ng for n = 2 donors (C) or at 10 μg for n = 6 donors (D). Error bars represent SEM of all data points for all donors. The Wilcoxon rank sum test was used to compare the number of IFN-γ spots between untransduced and transduced T cells (indicated by *). The Kruskal Wallis test was used to compare the number of IFN-γ spots between each antigen condition for untransduced or transduced cells. Any Kruskal Wallis test that had p < 0.05 was followed by post-hoc pairwise Wilcoxon rank sum tests and adjusted for multiple comparisons (indicated by #). All statistical comparisons are in Tables S1 and S2. *p < 0.05, **p < 0.005, ***p < 0.0005. ELISpot = Enzyme-linked immunosorbent spot; PHA = phytohemagglutinin; SEM = standard error of the mean.

To determine whether OM9.2-expressing T cells were capable of responding to the cognate 12mer/15mer peptide of ERV-K-Env, we repeated the ELISpot assay with T cells and free ERV-K-Env peptide (Fig. 2A). In general, nanogram amounts of peptide are used to test for antigen specificity of T cells [44] [45] [46] [47]. However, the original reported functional testing of this TCR was performed using relatively high concentrations of peptide—in the tens of micrograms range [36]. Therefore, we tested both a low concentration of peptide (200 ng per well, Fig. 2C) and a high concentration of peptide (10 μg per well, Fig. 2D). Again, both OM9.2 T cells and untransduced T cells from n = 6 donors had low background secretion of IFN-γ when cultured in the absence any antigen or with low or high concentrations of the negative control peptide actin (Fig. 2C–D). As an additional control, neither OM9.2 T cells nor untransduced T cells from donors 1 and 2 secreted IFN-γ in response to a low or high concentration of an HLA-mismatched off-target control peptide from pp65 (Fig. 2C–D). The epitope of pp65 used here is an HLA-B*35-restricted peptide from cytomegalovirus as donors 1–6 were HLA-B*35-negative (Table S3). OM9.2 T cells were not capable of responding to a low or high concentration the ERV-K-Env peptide as there was no significant difference in IFN-γ secretion in the presence of antigen compared to untransduced T cells (Fig. 2C–D). Again, all T cells were immunologically functional as they secreted IFN-γ in the presence of PHA. These data indicate that OM9.2 T cells are not capable of recognizing free ERV-K-Env peptide in solution, which led us to hypothesize that the affinity of the OM9.2 TCR for the cognate peptide was low.

To confirm this *in silico*, we ran the ERV-K-Env epitope sequence through the HLA-peptide prediction software NetMHCpan-4.1 [48] to obtain the predicted binding affinities of smaller peptides derived from the epitope to HLA-A*03:01. These smaller peptides are more likely to bind to HLA-A*03:01 with a high affinity than the entire 12mer/15mer epitope, which is longer than canonical HLA-I epitopes. The predicted binding affinities were all low, in the μM affinity range (Table S4). High TCR binding affinity for a peptide and a given HLA molecule is typically in the nM affinity range [49].

### OM9.2 T cells were not activated by the ERV-K-Env peptide when presented by peptide-pulsed APCs.

**Figure 3:**
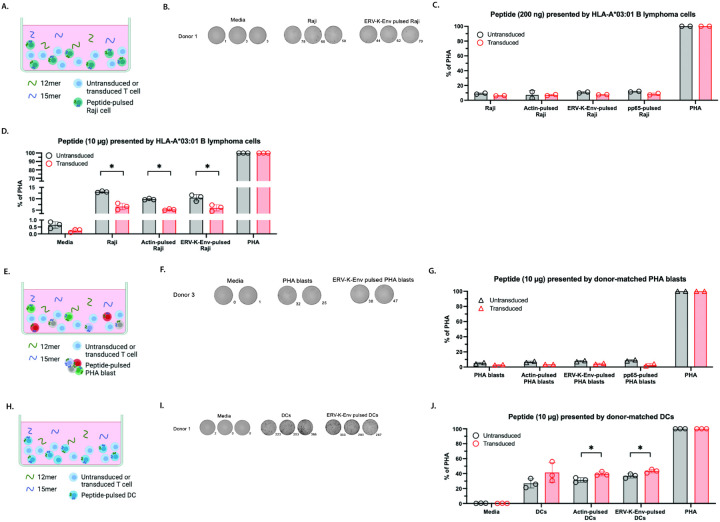
OM9.2 T cells were not activated by the ERV-K-Env peptide when presented by peptide-pulsed APCs. A, E, H. Diagrams of the ELISpot assay designs used in B-D, F-G, and I-J, respectively. B, F, I. Representative images of developed wells for OM9.2 T cell levels of background IFN-γ secretion (media) and IFN-γ secretion in the presence of unpulsed APCs and ERV-K-Env-pulsed APCs for the ELISpot assays quantified in C-D, G, and J, respectively. Spot numbers are indicated to the bottom right of each image. C-D, G, J. Number of IFN-γ spots per well normalized to the positive control (expressed as % of PHA) of untransduced T cells (black) and OM9.2 T cells (red) for the indicated conditions: Media = background level of IFN-γ secretion from T cells, Raji/PHA blast/DC = background level of IFN-γ secretion from T cells co-cultured with unpulsed APCs, actin-pulsed Raji/PHA blast/DC = negative control-pulsed APCs, ERV-K-Env-pulsed Raji/PHA blast/DC = ERV-K-Env-pulsed APCs, pp65-pulsed Raji/PHA blast/DC = HLA-B*35 off-target control pulsed APCs, PHA = positive control. Each donor is indicated by a different symbol. ELISpot conditions were plated with the indicated peptide concentrations in duplicate or triplicate as cell numbers allowed for n = 1 donor. Error bars represent SD of all data points for one donor. The Wilcoxon rank sum test was used to compare the number of IFN-γ spots between untransduced and transduced T cells (indicated by *). The Kruskal Wallis test was used to compare the number of IFN-γ spots between each antigen condition for untransduced or transduced cells. Any Kruskal Wallis test that had p < 0.05 was followed by post-hoc pairwise Wilcoxon rank sum tests and adjusted for multiple comparisons (indicated by #). All statistical comparisons are in Tables S1 and S2. *p < 0.05, **p < 0.005, ***p < 0.0005. APCs = antigen presenting cells; ELISpot = Enzyme-linked immunosorbent spot; PHA = phytohemagglutinin; SD = standard deviation.

To address this low affinity of the epitope to the TCR, we then used antigen presenting cells (APCs) in the ELISpot assays. Although free peptide is capable of binding to MHC molecules on T cells in the context of an ELISpot assay, T cells biologically recognize antigen best when it is presented by another cell [50]. To determine whether OM9.2 expressing cells could respond to the ERV-K-Env peptide when presented by a professional APC, we used the HLA-A*03:01 + Burkitt’s lymphoma B cell line Raji, which is latently infected with Epstein-Barr virus (EBV) [51] [52]. Raji cells were pulsed with peptides prior to plating the ELISpot assay, to allow for uptake and presentation of the peptide, as has been previously published [44] [45] (Fig. 3A). A higher level of IFN-γ secretion was observed from both OM9.2 T cells and untransduced T cells in the presence of unpulsed Raji cells (Fig. 3B–D, “Raji”, Figure S3A-C). The source of this increased IFN-γ secretion may be due to the HLA mismatching on the non-HLA-A*03:01 alleles between the T cells and the Raji cells, to T cell response to the EBV-infected Raji cells, or both [53] [54] [55]. However, compared to unpulsed Raji cells, OM9.2 T cells did not secrete more IFN-γ than untransduced T cells when cultured with any peptide-pulsed Raji cells at a low (200 ng) or high (10 μg) concentration of peptide (Fig. 3B–D, “antigen-pulsed Raji”). These data show that the OM9.2 TCR did not respond to low or high concentrations of the ERV-K-Env peptide when presented by peptide-pulsed HLA-A*03:01 + Raji cells.

We then evaluated whether transduced T cells could respond to the ERV-K-Env peptide when it was presented by artificially generated APCs derived from the T cell donors, for a fully HLA-matched system. We pulsed PBMCs from the same donors that the T cells were generated from with PHA to induce the formation of PHA-activated T cells (PHA blasts) (Fig. 3E). PHA blasts express high levels of HLA I [56]. Despite the complete HLA matching of T cells to APCs, OM9.2 T cells still had low background secretion of IFN-γ when cultured with unpulsed PHA blasts or any peptide-pulsed PHA blasts compared to untransduced T cells (Fig. 3F–G, n = 1 donor, **Figure SD-E**) even in the presence of a high concentration of antigen. These data indicate that OM9.2 T cells did not respond to a high concentration of the ERV-K-Env peptide even when presented by HLA matched artificially generated APCs.

Next, we shifted from using artificially generated APCs to using the most potent, professional APCs: dendritic cells (DCs). We matured monocyte-derived DCs from PBMCs from the same donor, as previously published [57], and repeated the experiment from Fig. 3E–G, but with DCs instead of PHA blasts (Fig. 3H). Again, we observed low secretion of IFN-γ when the OM9.2-expressing T cells were cultured with unpulsed DCs or any peptide-pulsed DCs compared to untransduced T cells (Fig. 3I–J, **Figure S3F-G**). The high background level of IFN-γ is likely due to IFN-γ produced by DCs in response to the lipopolysaccharide added in the maturation protocol [58]. Together, these data indicate that OM9.2-expressing T cells were not capable of responding to the ERV-K-Env peptide when presented by HLA matched artificially generated APCs or professional APCs.

### OM9.2 T cells were activated by a high concentration of the ERV-K-Env peptide when presented by B LCLs derived from donor OM9.

**Figure 4:**
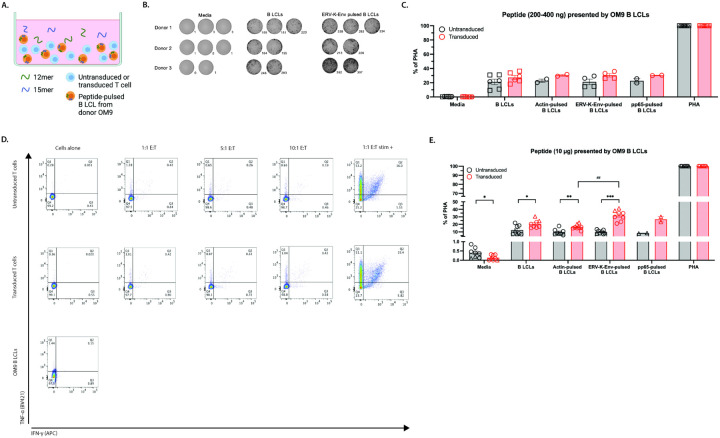
OM9.2 T cells were activated by a high concentration of peptide-pulsed B LCLs. A. Diagram of the ELISpot assay design used in B-C, and E. B. Representative images of developed wells for OM9.2 T cell levels of background IFN-γsecretion (media) and IFN-γ secretion co-cultured with unpulsed and peptide-pulsed OM9-derived B LCLs for the ELISpot assays quantified C and E. Spot numbers are indicated to the bottom right of each image. C., E. Number of IFN-γ spots per well normalized to the positive control (expressed as % of PHA) of untransduced T cells (black) and OM9.2 T cells (red) for the indicated conditions: Media = background level of IFN-γ secretion from T cells, B LCLs = background level of IFN-γ secretion from T cells co-cultured with unpulsed B LCLs, actin-pulsed B LCLs = negative control-pulsed B LCLs, ERV-K-Env-pulsed B LCLs, pp65-pulsed B LCLs= HLA-B*35 off-target control pulsed B LCLs, PHA = positive control. Each donor is indicated by a different symbol. ELISpot conditions were plated with the indicated peptide concentrations in duplicate or triplicate as cell numbers allowed for n = 2 donors (C) or n = 3 donors (E). The Wilcoxon rank sum test was used to compare the number of IFN-γ spots between untransduced and transduced T cells (indicated by *). The Kruskal Wallis test was used to compare the number of IFN-γ spots between each antigen condition for untransduced or transduced cells. Any Kruskal Wallis test that had p < 0.05 was followed by post-hoc pairwise Wilcoxon rank sum tests and adjusted for multiple comparisons (indicated by #). All statistical comparisons are in Tables S1 and S2. *p < 0.05, **p < 0.005, ***p < 0.0005. D. Flow cytometry plots of intracellular IFN-γ and TNF-α from indicated cell types after 6 hours of co-culture. Cells were plated in a single well for one technical replicate. Gating strategy in Figure S4C. ELISpot = Enzyme-linked immunosorbent spot; B LCL = B lymphoblastoid cell line; PHA = phytohemagglutinin; SEM = standard error of the mean.

As a final test to determine whether the OM9.2 T cells could respond to the ERV-K-Env peptide, we used professional APCs derived from the original HIV-1 + HLA-A*03:01 + donor that the TCR was isolated from (donor OM9). There was also a high background level of IFN-γ secretion by OM9.2 T cells and untransduced T cells in response to the OM9 B LCLs (Fig. 4A–C, n = 2 donors, **Figure S3H-J**). Similar to the results observed with the Raji cells (Fig. 3B–D), it cannot be determined whether this higher background level is due to the HLA mismatching on the non-HLA-A*03:01 alleles between the T cells and the OM9 B LCLs, to T cell response to the EBV-infected B LCLs, or both. Despite the elevated background signal to the OM9 B LCLs, OM9.2 T cells did not secrete significantly more IFN-γ than untransduced T cells when cultured with unpulsed B LCLs or OM9 B LCLs pulsed with 200 ng of peptide (Fig. 4C). To confirm our IFN-γ ELISpot findings, we also examined the response of OM9.2 expressing T cells co-cultured with B LCLs via intracellular cytokine staining. OM9.2-expressing T cells showed minimal staining for either IFN-γ or another pro-inflammatory cytokine TNF-α, when co-cultured with unpulsed B LCLs for either 6 hours or 24 hours at several effector-to-target-cell ratios (Fig. 4D and **Figures S4-S5**).

However, in the presence of a high concentration of ERV-K-Env peptide, there was significantly more IFN-γ secreted by OM9.2 T cells in response to ERV-K-Env-pulsed OM9 B LCLs compared to untransduced T cells (Fig. 4E, p = 0.0008). We also observed a significant increase in the amount of IFN-γ secreted by OM9.2 T cells in response to ERV-K-Env-pulsed OM9 B LCLs compared to actin-pulsed OM9 B LCLs (Fig. 4E, p = 0.0066). Although a significant increase in the amount of IFN-γ secreted by OM9.2 T cells in media alone and in response to unpulsed OM9 B LCLs and actin-pulsed OM9 B LCLs compared to untransduced cells was observed, these increases are smaller than the increase observed for the ERV-K-Env peptide add likely have minimal biological significance (Fig. 4E, p = 0.015, p = 0.046, p = 0.0087, respectively). As the OM9.2-expressing T cells only responded functionally to the ERV-K-Env peptide when it was present at a high concentration, these results support a) the bioinformatic predictions that the HLA-A*03:01-restricted ERV-K-Env TCR possesses a low affinity for the ERV-K-Env peptide and b) the findings from the paper where this TCR was originally identified [36].

### OM9.2 T cells were activated by a high concentration of the ERV-K-Env peptide when presented by peptide-pulsed B LCLs derived from a different HLA-A*03:01 donor.

**Figure 5:**
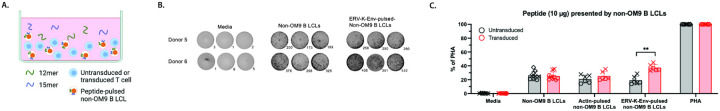
OM9.2 T cells were activated by a high concentration of peptide-pulsed B LCLs derived from another donor. A. Diagram of the ELISpot assay design used in B-C. B. Representative images of developed wells of OM9.2 T cell levels of background IFN-γ secretion (media) and IFN-γ secretion in the presence of unpulsed non-OM9 B LCLs and ERV-K-Env-pulsed non-OM9 B LCLs for the ELISpot assays quantified in C. Number of spots in each well are indicated to the bottom right of each image. C. Number of IFN-γ spots per well normalized to the positive control (expressed as % of PHA) of untransduced T cells (black) and OM9.2 T cells (red) for the indicated conditions: Media = background level of IFN-γ secretion from T cells plated alone, Non-OM9 B LCLs = background level of IFN-γ secretion from T cells in the presence of unpulsed non-OM9 B LCLs,actin-pulsed non-OM9 B LCLs = negative control pulsed non-OM9 B LCLs, ERV-K-Env-pulsed non-OM9 B LCLs, PHA = positive control. Each donor is indicated by a different symbol. ELISpot conditions were plated with the indicated peptide concentrations in duplicate or triplicate as cell numbers allowed for n = 2 donors. Error bars represent SEM of all data points for all donors. The Wilcoxon rank sum test was used to compare the number of IFN-γ spots between untransduced and transduced T cells (indicated by *). The Kruskal Wallis test was used to compare the number of IFN-γ spots between each antigen condition for untransduced or transduced cells. Any Kruskal Wallis test that had p < 0.05 was followed by post-hoc pairwise Wilcoxon rank sum tests and adjusted for multiple comparisons (indicated by #). All statistical comparisons are in Tables S1 and S2. *p < 0.05, **p < 0.005, ***p < 0.0005. ELISpot = Enzyme-linked immunosorbent spot; B LCL = B lymphoblastoid cell line; PHA = phytohemagglutinin; SEM = standard error of the mean.

We then wanted to determine whether the ability of the OM9.2 T cells to respond to the ERV-K-Env peptide when presented by OM9 B LCLs extended to B LCLs from generated from another HLA-A*03:01 donor. This donor was HIV-1 seronegative, unlike donor OM9. We repeated the ELISpot assay with a high concentration of ERV-K-Env peptide and again observed a significant increase in IFN-γ secreted by OM9.2 T cells in response to ERV-K-Env-pulsed non-OM9 B LCLs compared to untransduced cells (Fig. 5A–C, p = 0.004, n = 2 donors, **Figure S3K-L**). These data indicate that OM9.2 T cells can respond to a high concentration of the ERV-K-Env peptide when it is presented by HLA-A*03:01 + B LCLs.

## DISCUSSION

The ability of the OM9.2 TCR to recognize and kill HIV-1-infected cells, coupled with the defined expression of ERVs in various cancers, including OC, suggests that this TCR may be repurposed to treat patients with HLA-matched solid tumors. However, we found that the ability of the ERV-K-Env TCR to recognize and kill HIV-1-infected cells does not translate well to the setting of healthy HIV-1-seronegative donors, suggesting that the ERV-K-Env peptide is not an immunogenic tumor-associated antigen. Here, we demonstrated that T cells derived from healthy donors transduced with the OM9.2 TCR were only capable of being activated by the cognate peptide when it was a) present at high concentrations and b) presented by peptide-pulsed HLA-A*03:01 B LCLs. These results agree with the findings where this T cell clone was originally identified [36]. In that study, the ERV-K-Env-specific CD8 + T cell clone derived from donor OM9 secreted IFN-γ in response to high concentrations (tens of micrograms amounts) of the ERV-K-Env epitope in the presence of B LCLs derived from donor OM9, as we observed in our study. In contrast, tumor-specific T cells are typically able to secrete IFN-γ in response to their cognate peptides at concentrations as low as a few hundred nanograms without professional APCs present [59] [60]. The high concentration of peptide required to elicit a T cell response suggests that the OM9.2 TCR is of low affinity for the cognate peptide.

While the results in our study confirm those of Jones *et al,* they conflict with the findings from Rycaj *et al.,* in which T cells expanded from patients with OC were able to secrete IFN-γ in response to ERV-K-Env [27]. A few key differences between our study and the Rycaj *et al*. study may account for the disparate findings. One major difference is that Rycaj *et al*. used the entire ERV-K-Env protein as the antigen, rather than the minimal 12mer/15mer epitope used in our study. It is likely that the epitope (or epitopes) eliciting an IFN-γ response from the T cells in the Rycaj *et al*. study is not the epitope used in our study. Another difference from the Rycaj *et al*. study is that T cells were expanded against ERV-K-Env from patients with OC while our study used T cells isolated from healthy donors. Although T cells transduced with the ERV-K-Env TCR should recognize the epitope regardless of the donor, this difference lends further support to the hypothesis that the ERV-K-Env epitope used in this study is not contributing to the IFN-γ response seen in the Rycaj *et al*. study. Another publication from the same group also demonstrated IFN-γ secretion from T cells expanded from patients with breast cancer, but not T cells expanded from healthy donors [61]. When combined with our data, these findings suggest that even though the HLA-A*03:01-restricted epitope may not be a strong tumor-associated antigen, other portions of the ERV-K-Env surface domain may be.

As the only professional APC capable of eliciting a T cell response to the ERV-K-Env peptide is B LCLs, this suggests that latent infection of professional APCs may somehow be facilitating alternative processing and presentation of this epitope. While we initially concluded that the seropositivity of the OM9 donor for HIV-1 + could be contributing to the ability of the OM9 B LCLs to present the antigen in a way that is recognized by this TCR, the data with the non-OM9 B LCLs suggest that HIV-1 seropositivity does not contribute to the B LCLs’ ability to present this antigen to T cells. Instead, the latent infection of B LCLs with EBV might be contributing to the ability of these APCs to present the ERV-K-Env peptide efficiently. It is worth noting that although we saw no response from OM9.2 T cells to the peptide presented by Raji cells, these cells are also latently infected with EBV [53] [54]. However, the malignant state of Raji cells may contribute to their inability to present this peptide. Still the possibility of only latently infected cells being able to present this unusually long HLA-A*03:01-restricted peptide cannot be ruled out by the experiments in this work alone.

Some elements of our study design limit the conclusions we can draw from our data. First, we used the level of eGFP expression as a surrogate marker of the ERV-K-Env TCR expression. While the use of a tetramer would have directly confirmed surface expression of the TCR, discussions with commercial vendors concluded that generating an MHC I tetramer with a 12- or 15-amino acid peptide in the peptide-binding groove would not be feasible. However, the replication of the results from this study with the Jones *et al*. study suggest that the TCR was expressed on the surface of the transduced T cells.

Additionally, the high background level of IFN-γ secretion by the T cells in the presence of some APCs—in particular Raji cells, DCs, and B LCLs—made it difficult to determine if the T cells recognized the ERV-K-Env peptide. However, the high concentration of the ERV-K-Env peptide used in Figs. 4 and 5 resulted in sufficient activation of OM9.2 T cells to be observed even in the presence of this high background signal. The data from these experiments and the controls (**Figure S3**) suggest that the signal seen in Figs. 4 and 5 is not just background IFN-γ secretion upon recognition of APCs.

This study provides evidence that the ERV-K-Env epitope is likely not a strong targetable tumor-associated antigen in OC due to a) no increased response from OM9.2 T cells in the presence of HLA-A*03:01 OC cells compared to untransduced T cells and b) an increased response from OM9.2 T cells only in the presence of high concentrations of the ERV-K-Env peptide when presented by peptide-pulsed HLA-A*03:01 APCs. However, many recent reports have shown that RE-derived antigens are presented by MHC on tumor cells [62] [63] [64] [65]. While these studies confirm the existence of RE-derived antigens presented by tumor cells, it remains to be determined whether T cells can be reliably generated to recognize and respond to these antigens. Recent data has also identified antibodies reactive to the ERV-K envelope glycoproteins in patients with lung cancer, suggesting that while ERV-K-Env may be immunogenic, it might not be an optimal T cell target [66]. The same study showed increased titers of antibodies recognizing ERV-K envelope glycoproteins in these patients following the administration of immune checkpoint blockade. As patients with OC do not respond well to immune checkpoint blockade, the additional targeting of ERV-K-Env by antibodies may boost immune recognition of ovarian tumors. This treatment strategy might also be effective with the combination of epigenetic inhibitors and antibodies recognizing ERV-K-Env. Another approach may be to target chimeric RE-derived transcripts, which derive from cryptic promoters within REs [62]. A pan-cancer analysis of over 30 tumor types in The Cancer Genome Atlas have shown these RE-derived chimeric transcripts to be immunogenic antigens. These RE-derived antigens may boost immune recognition of tumors, thus resulting in increased control of disease over time and ultimately, longer survival for patients with malignancies, both with OC and beyond.

## CONCLUSIONS

This study demonstrates that the ERV-K-Env-specific TCR is likely not a suitable immunogenic tumor-associated antigen for OC. The cognate epitope was only recognized at high concentrations when presented by HLA-A*03:01 B LCLs. Future efforts to develop RE-derived T cells for OC should focus on other targets, in particular chimeric RE-derived peptides.

## METHODS

Aim: To determine the efficacy of the ERV-K-Env TCR to recognize its cognate epitope as a therapeutic option for OC

Design: Use of *in vitro* experiments to transduce cells from primary healthy donors and measure T cell functionality

Setting: *In vitro*

### Plasmid preparation.

The OM9.1 ERV-K-Env TCR plasmid construct was a kind gift from Dr. Mario Ostrowski. The OM9.1 construct was further optimized (construct OM9.2) by cloning it into a lentiviral vector under the control of the EF-1α promoter and confirmed by Sanger sequencing. For Donors 1–6, construct OM9.2 was transformed into Agilent XL1 Blue Supercompetent cells (Agilent, Cat. #200236), according to the manufacturer’s instructions. Plasmid pMD2.G (Addgene, Cat. #12259) was transformed into 5-alpha competent E. coli cells (NEB, Cat. #C2987I), according to the manufacturer’s instructions. Plasmids pRSV.Rev (Addgene, Cat. #12253) and pMDLg/RRE (Addgene, Cat. #12251) were obtained as agar stabs. For Donors A-C, the OM9 TCR and packaging plasmids (all from Dr. Mario Ostrowski) were transformed into 5-alpha competent E. coli cells (NEB, Cat. #C2987I) according to the manufacturer’s instructions. Plasmids were isolated from bacterial pellets using the PureLink HiPure Plasmid Midiprep kit (Invitrogen, Cat. #K210004) according to the manufacturer’s instructions.

### Cell sources.

We have received the following cell lines from Dr. Dennis Slamon’s laboratory: ES-2 and TOV112D. Cell lines were tested using a short tandem repeat (STR) profiling service provided by the Johns Hopkins Genetic Resources Core Facility (GRCF). STR profiling by the GRCF is carried out following the ANSI/ATCC ASN-0002–2011, Authentication of Human Cell Lines: Standardization of STR Profiling. For cell lines derived from repositories, the profile of the cell line is used for comparison using verification tools found on the repository web sites to determine relatedness of the line to those held by the repositories. GRCF uses the following databases to generate the reports: Leibniz Institute DSMZ German Collection of Microorganisms and Cell Cultures, American Tissue Culture Collection, Japanese Collection of Research Bioresources. The algorithms used in the American National Standards Institute (ANSI) and the American Type Culture Collection Standards Development Organization’s (ATCC SDO) report ANSI/ATCC ASN-0002–2011 are used to calculate percent match or evaluation value (EV) which were then reported to our lab. 293T cells (Cat. #CRL-3216), SupT1 cells (Cat. #CRL-1942), and Raji cells (Cat. #CCL-86) were purchased from ATCC. B LCLs were kind gifts from Dr. R. Brad Jones (OM9 B LCLs) and Dr. Catherine M. Bollard (non-OM9 B LCLs). In general, B lymphoblastoid cells (B LCLs) were generated by immortalizing B cells from donor OM9 with the B95–8 Epstein-Barr virus according to published protocols [42]. De-identified healthy donor peripheral T cells were purchased from the Human Immunology Core at the University of Pennsylvania, de-identified commercially available buffy coats were purchased from New York Blood Center (New York, NY), and de-identified platelet filters were purchased from Oklahoma Blood Institute (Oklahoma City, NY). Cells were tested for mycoplasma yearly using the MycoAlert^®^ Kit (Lonzo, Cat #LT07–703).

### Lentiviral transduction of T cells.

#### Lentivirus production.

293T cells were cultured in DMEM (Corning, Cat. #10–013-CV) + 10% FBS (R&D Systems, Cat. #S12450H) + 1% penicillin/streptomycin (Gibco, Cat. #15070063). On the day of transfection, the media was replaced with T cell media (RPMI [Corning, Cat. #10–040-CV] + 10% FBS + 1% penicillin/streptomycin + 1X MEM non-essential amino acids (MEM NEAA) [Corning, Cat. #25–025-CI] + 1X GlutaMAX [Gibco, Cat. #35050–061] + 10 mM HEPES [Gibco, 15630–080]). Lentivirus was made by combining the appropriate plasmids with OptiMEM (Gibco, Cat. #11058–021), adding the plasmid mixture to 293T cells, and harvesting at 48 hours by filtering through a 0.45 μm PVDF filter (CellTreat, Cat. #229745).

### Activation and lentiviral transduction of T cells.

Isolated T cells were activated with anti-CD3/anti-CD28 Dynabeads (Gibco, Cat. #11131D) and 30 U/mL recombinant human IL-2 (Peprotech, Cat. #200–02). T cells were plated and rested overnight at 37°C prior to transduction. T cells were transduced with lentivirus (construct OM9.2 for SupT1 cells and Donors 1–6 and construct OM9.1 for Donors A-C) and transduction efficiency was measured by detection of eGFP-fluorescence using a BD LSR Fortessa, a BD FACS Celesta, or a BD Influx cytometer.

### Interferon-γ ELISpot.

Wells of a PVDF-membrane plate (Millipore Sigma, Cat. #MSIPS4W10) were activated with 70% ethanol then coated with 1 μg of anti-human-IFN-γ antibody 1-D1K (Mabtech, Cat. # 3420-3-1000, RRID #AB_907282). The plate was incubated at room temperature for at least 4 hours or overnight at 4°C. Antigens were plated at 200–400 ng per well for low concentration and 10 μg per well for high concentration. Phytohemagglutinin (PHA, ThermoScientific Cat. #R30852801) was plated at 1–5 μg per well. Cells were plated at 1×10^**5**^ cells/well (co-culture conditions contained 1×10^**5**^ cells of each type) in duplicate or triplicate as cell numbers allowed. The plate was incubated overnight at 37°C. Spots were developed with 0.1 μg of biotinylated anti-IFN-γ 7-B6–1 antibody (Mabtech, Cat. #3420-6-1000, RRID #AB_907273), followed by the Vectastain Elite ABC-HRP Kit (Vector Laboratories, Cat. #PK-6100), and 3-amino-9-ethylcarbazole (Sigma Aldrich, Cat. #A6926). Wells were imaged and counted using an AID vSpot Spectrum plate reader (Autoimmun Diagnostika GMBH).

### Flow cytometry and intracellular cytokine staining.

Cells were stained with Live/dead Fixable Aqua (Invitrogen, Cat. #L34965) and the antibodies diluted to titrated amounts indicated in Table 1 in Facs buffer (PBS + 1% FBS). For intraceullar cytokine staining, B and T cells were plated alone or in co-culture at a ratio of 1:1, 5:1, or 10:1 in T cell media (RPMI + 10% FBS + 1% penicillin/streptomycin + 1X MEM NEAA + 1X GlutaMAX + 1 mM HEPES) with 100 U/mL IL-2 and incubated at 37°C. For the 6-hour conditions, 1X protein transport inhibitors (Invitrogen, Cat. #00-4980-93) or 1X cell stimulation cocktail (Invitrogen, Cat. #00-4970-93) were added after 1 hour of co-culture, then cells were incubated at 37°C for 5 hours. For the 24-hour conditions, 1X protein transport inhibitors or 1X cell stimulation cocktail were added at 20 hours of co-culture, then cells were incubated at 37°C for 4 hours. Cells were stained with the viability marker listed above, the surface antibodies diluted to titrated amounts indicated in Table 2, fixed in Cytofix/Cytoperm solution (BD, Cat. #51–2090KZ), and permeabilized with 1X permeabilization/wash buffer (BD, Cat. #51–2091KZ). Permeabilized cells were stained with appropriate diluted antibodies or 1X permeabilization buffer. Data was acquired using a BD FACS Celesta and analyzed using FlowJo v10.8.1 with manual compensation using single stained cells and UltraComp eBeads (ThermoFisher, Cat. #01-2222-41).

### Statistics.

Statistics were performed in RStudio v4.0.1. The Wilcoxon rank sum test was used to compare the number of IFN-γ spots between untransduced and transduced T cells (indicated by *). The Kruskal Wallis test was used to compare the number of IFN-γ spots between each antigen condition. Any Kruskal Wallis test that had p < 0.05 was followed by post-hoc pairwise Wilcoxon rank sum tests and adjusted for multiple comparisons using the Bonferroni correction (indicated by #). Values from all statistical comparisons are in Tables S1 and S2. *p < 0.05, **p < 0.005, ***p < 0.0005.

## Figures and Tables

**Table 1:** Surface antibodies used in flow cytometry experiment. Vendor, catalogue, and titration information for surface antibodies used in flow cytometry experiments (Figure S2C).

Marker	Cellular location	Fluorophore	Supplier	Catalogue #	μL antibody per million cells
CD3	Surface	BV605	Biolegend	317322	1
TCR-α/β	Surface	APC	Biolegend	306718	1

**Table 2:** Surface antibodies used in intracellular cytokine staining. Vendor, catalogue, and titration information for surface and intraceullular antibodies used in flow cytometry experiments (Fig. 4D and **Figure S5**).

Marker	Cellular location	Fluorophore	Supplier	Catalogue #	μL antibody per million cells
CD3	Surface	PerCP/Cy5.5	Biolegend	300328	0.625
CD19	Surface	APC/Cy7	Biolegend	363010	1.25
HLA-A*03	Surface	PE	Invitrogen	12-5754-42	0.625
IFN-γ	Intracellular	APC	Biolegend	502512	5
TNF-α	Intracellular	BV421	Biolegend	502932	2.5

## Data Availability

All data are contained in the main article and the supplementary information.

## Abbreviations

RE: repetitive element
OC: ovarian cancer
ERV-K: Env endogenous retrovirus K *envgene*
HIV-1: human immunodeficiency virus-1
PBMC: peripheral blood mononuclear cell
HLA: human leukocyte antigen
MHC: major histocompatibility complex
IFN-γ: interferon-y
APC: Antigen presenting cell
EBV: Epstein Barr Virus
DC: dendritic cell
B LCL: B lymphoblastoid cell line
